# CA19-9-Producing early gastric adenocarcinoma arising in hyperplastic foveolar polyp: a very unique resection case

**DOI:** 10.1186/1746-1596-6-112

**Published:** 2011-11-10

**Authors:** Xin Guo, Sohsuke Yamada, Haruki Omori, Ke-Yong Wang, Takashi Tasaki, Atsunori Nabeshima, Kimitoshi Kohno, Yasuyuki Sasaguri

**Affiliations:** 1 Department of Pathology and Cell Biology, University of Occupational and Environmental Health, Kitakyushu 807-8555, Japan; 2 Department of Internal Medicine, Iwami Clinic, Masuda 698-0024, Japan; 3 Department of Molecular Biology, School of Medicine, University of Occupational and Environmental Health, Kitakyushu 807-8555, Japan

**Keywords:** CA19-9, CA19-9-producing adenocarcinoma, early cancer, hyperplastic foveolar polyp, stomach

## Abstract

Here we report the first case of carbohydrate antigen (CA) 19-9-producing early gastric adenocarcinoma arising in polyp. A solitary pedunculated polyp lesion of the stomach, measuring 26 × 20 × 20 mm, was noticed in a 76-year-old Japanese woman due to an abdominal disorder, associated with a markedly high serum CA19-9 level (2,172.6 U/ml). After endoscopic mucosal resection was performed, the CA19-9 level was drastically decreased and normalized. The scanning view of immunohistochemical staining of CA19-9 exhibited a focal, not diffuse, positive-expression in the hyperplastic epithelium and, especially, in the irregular and fused tubular glands and the mucinous material secreted into the dilated glands. In particular, microscopic examination of the strongly CA19-9-positive areas showed structurally atypical epithelium containing mildly to focal moderately enlarged nuclei and prominent nucleoli with loss of cellular polarity, estimated as adenocarcinoma. No stromal invasion was evident. Immunohistochemically, distinct nuclear stainings for p53 and Ki-67 were seen, occasionally conforming to the CA19-9-positive atypical cells, respectively, confirmed by double immunostaining. These hyperplastic and atypical cells were classified into the pure gastric phenotype by mucin histochemical methods. Based on these features, we finally made a conclusive diagnosis of CA19-9-producing *in situ *well differentiated adenocarcinoma of gastric type arising in hyperplastic foveolar polyp. We suggest that the markedly high serum CA 19-9 level could be indicative of carcinoma in polyp at the very least.

**Virtual Slides:**

The virtual slides for this article can be found here: http://www.diagnosticpathology.diagnomx.eu/vs/1692254825554310

## Background

It is generally accepted that malignant transformation and progression in tumors are closely related to alterations in cell-surface carbohydrate antigens (CAs) with frequent aberrant glycosylation [1]. CA19-9 is a sialylated-Lewis^a ^blood group antigen which has been shown to be useful for monitoring malignant tumor status, including the invasive and/or metastatic behavior of carcioma cells affecting adhesion, motility or immunogenicity [2,3]. Monoclonal antibody 19-9, derived from mice spleen cells immunized with human colon adenocarcinoma cell line SW1116, can detect its antigen in the serum of patients, mainly existing in mucins containing a sialylated lacto-*N*-fucopentaose II, IV^3^-α-NeuNAc-III^4^-α-Fuc-LcOse_4_, in which LcOse_4 _is Galβ1-3GlcNAcβ1-3Galβ1-4Glc [4,5]. The existence of CA19-9-producing gastric cancer has been suggested, defined by three factors according to a Japanese report: first, the patients have high serum CA19-9 levels before resection; second, CA19-9 expression can be identified by using the CA19-9 antibody in the resected specimens; third, the serum CA19-9 levels are significantly decreased after resection [6]. At least 28 cases of CA19-9-producing gastric cancer have been reported in the Japanese literature, but none in the English language literature [6]. Here we report an extremely rare and the first case of a CA19-9-producing gastric adenocarcinoma arising in hyperplastic foveolar polyp.

## Case presentation

**Figure 1:**
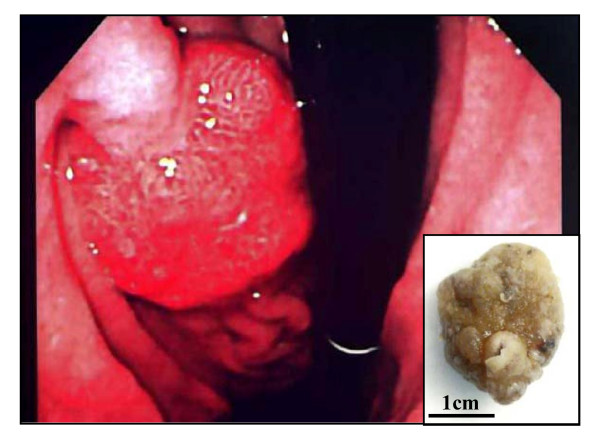
**Endoscopic and gross examination of the polyp**. Gastrointestinal endoscopy shows a solitary pedunculated polyp lesion, classified as Yamada type IV, in the posterior wall of lower gastric body. On gross finding of the resected specimen (inset), the surface of this polyp, measuring 26 × 20 × 20 mm, reveals focally irregular features with erosion and hemorrhage.

The patient was a 76-year-old woman, who had only hypertension with long-term administration of depressors in her past medical history. She had never taken Sucralfate, a drug for protection against gastric mucosa. She complained about abdominal disorder at the rt. lower portion, but nothing remarkable was found in her laboratory data except for a markedly high serum CA19-9 level (2,172.6 U/ml, normal ranges: < 37 U/ml) by electrochemiluminescence detection method for development of immunoassays (ECLIA; BECKMAN COULTER, Tokyo, Japan). The other tumor markers, such as carcino-embryonic antigen (CEA) or CA125, were within normal limits. No remarkable change was seen in the abdominal and chest CT. Gastrointestinal endoscopy was performed for further investigation, revealing a solitary pedunculated polyp lesion measuring approximately 25 mm in the posterior wall of the lower gastric body (Figure 1). It was estimated as type IV according to Yamada's classification [7]. Eleven days after endoscopic mucosal resection, the CA19-9 level was drastically decreased (136.4 U/ml). Moreover, it was down to 60 U/ml 1 day after appendectomy due to acute appendicitis (13 days after the resection), and was within the normal limit 1 year later. The patient is alive and well without recurrence.

## Pathological findings

**Figure 2:**
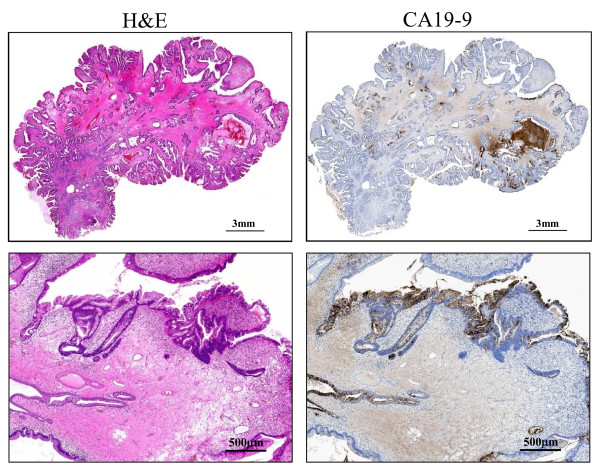
**Scanning view and low-power image of the polyp**. **(H & E) **The scanning view and low-power image of Hematoxylin-eosin (H & E) staining demonstrate that the lesion is covered by hyperplastic foveolar-type epithelium with focally distorted or cystically dilated change, supported by abundant edematous fibrovascular stroma without apparent invasive components (left upper), and small irregular and fused tubular areas are found in the hyperplastic polyp (left lower). **(CA19-9) **The scanning view and lower-power image of immunohistochemical staining of CA19-9 on its sequential section show a focal, not diffuse, positive-expression in the hyperplastic epithelium and, especially, in the irregular and fused tubular glands (right lower) and the secreted mucinous material into the dilated glands (right upper).

**Figure 3:**
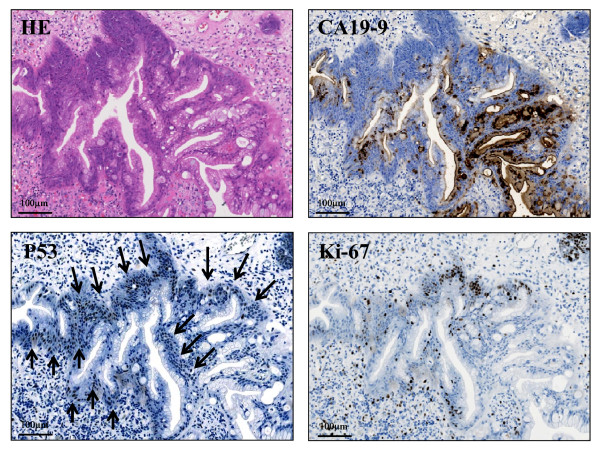
**Microscopic examination of the adenocarcinoma arising in polyp by hematoxylin-eosin (H & E) and immunohistochemical stainings**. **(H & E) **Small adenocarcinoma areas in the hyperplastic polyp show irregular and fused tubular structures, associated with surface erosion, granulation, hemorrhage, and inflammatory infiltrate. These structurally atypical epithelial cells contain mildly to focal moderately enlarged nuclei and occasionally prominent nucleoli with loss of cellular polarity. **(CA19-9) **These atypical glands are immunohistochemically positive for CA19-9, associated with strong expression especially in the secreted mucin. The CA19-9-positive staining displays not only a cytoplasmic but also an apical pattern. **(p53) **Distinct nuclear staining for p53 is occasionally conforming to these atypical cells (arrows). **(Ki-67) **Moreover, Ki-67 (MIB-1) labeling index is high (more than 5% compared to hyperplastic polyps), conforming to the small adenocarcinoma areas.

**Figure 4:**
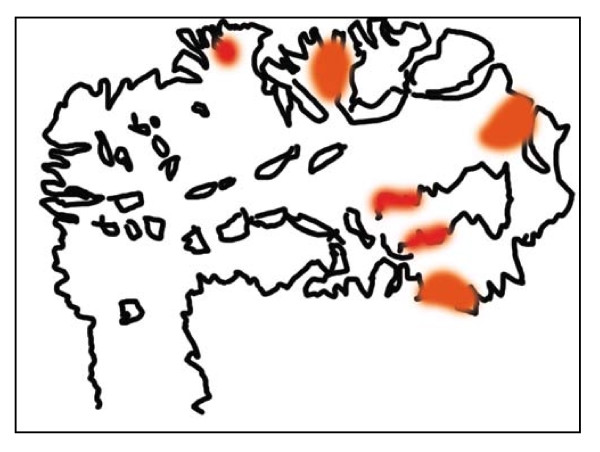
**Schematic map of the adenocarcinoma arising in polyp**. The present case has been diagnosed as CA19-9 producing gastric adenocarinoma arising in hyperplastic foveolar polyp. The small carcinoma components (red areas) arise in the surface of the polyp without apparent stromal invasion. Left lower side is a stalk of the adenocarcima in polyp.

**Figure 5:**
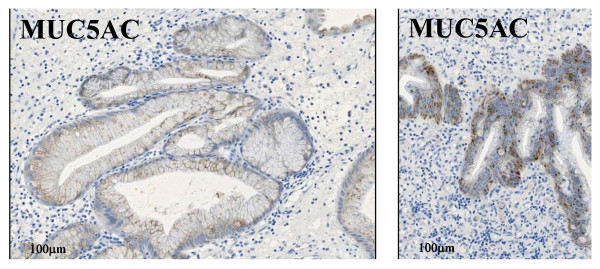
**Microscopic examination of the adenocarcinoma arising in polyp by mucin immunohistochemical methods**. Not only the hyperplastic (left) but the atypical (right) epithelium is also positive for MUC5AC in immunohistochemistry whereas negative for MUC2 or CD10, classified into pure gastric phenotype.

A gross examination of the resected specimen (Figure 1, inset) showed a polyp measuring 26 × 20 × 20 mm, the surface of which was focally irregular with erosion and hemorrhage. The scanning view (Figure 2, left upper) and low-power image (Figure 2, left lower) of Hematoxylin-eosin (H & E) staining demonstrated that the lesion was covered by hyperplastic foveolar epithelium with focally distorted, cystically dilated, or atypical change, supported by abundant edematous fibrovascular stroma without apparent invasive components. Additionally, the scanning view (Figure 2, right upper) and lower-power image (Figure 2, right lower) of immunohistochemical staining of CA19-9 (Dako Cytomation Co., Glostrup, Denmark, diluted 1:30) on its sequential section exhibited a focal, not diffuse, positive-expression in the hyperplastic epithelium and, especially, in the irregular and fused tubular glands and the mucinous material secreted into the dilated glands. Microscopic examination of the strongly CA19-9-positive small areas showed irregular and fused tubular structures, associated with surface erosion, granulation, hemorrhage, and inflammatory infiltrate (Figure 3). These structurally atypical epithelial cells contained mildly to focal moderately enlarged nuclei and occasionally prominent nucleoli with loss of cellular polarity (Figure 3). The CA19-9-positive staining displayed not only a cytoplasmic but also an apical pattern (Figure 3). Moreover, distinct nuclear staining for p53 (Dako Cytomation Co., Glostrup, Denmark, diluted 1:30) was occasionally conforming to the CA19-9-positive atypical cells (Figure 3), and we confirmed it by double immunostaining (CA19-9 with Vulcan Fast Red, and p53 with DAB as a substrate, respectively) of MACH 2 Double Stain system (Biocare Medical, LLC., Concord, CA, USA), revealing the distribution of both CA19-9- and p53-positive cells (data not shown). Additionally, the Ki67 (MIB-1; Dako, diluted 1:50) labeling index was high, more than 5% (Figure 3) compared to hyperplastic polyps in immunohistochemistry, conforming to the CA19-9-positive areas. Similarly, double immunostaining was performed to detect the distribution of both CA19-9- and Ki-67-positive cells (CA19-9 with DAB as a substrate, and Ki-67 with Vulcan Fast Red, respectively) by MACH 2 Double Stain system (Biocare Medical) (data not shown). These features indicated that the irregular small areas were not within regenerative atypia, but estimated as adenocarcinoma. No stromal invasion or vessel permeation was evident, and surgical margin was free of atypical cells. The schematic map of this adenocarcinoma arising in hyperplastic foveolar polyp is summarized in Figure 4. On the other hand, not only the hyperplastic but the atypical epithelium was also classified into pure gastric phenotype according to the immunopositivity of MUC5AC (Novocastra, Newcastle, UK, diluted 1:100) (Figure 5) and the immunonegativity of MUC2 (Novocastra, diluted 1:100) or CD10 (Leica, Newcastle, UK, diluted 1:20) by the mucin histochemical methods previously described by Yao and Kabashima, *et al *[8]. Based on all these findings, we finally made a conclusive diagnosis of CA19-9-producing well differentiated adenocarcinoma of gastric type arising in hyperplastic foveolar polyp.

## Discussion

It is well known that gastric hyperplastic polyps rarely demonstrate malignant transformation, the incidence of which is reported to be 0.8% to 4.5% [9,10]. The characteristics of the adenocarcinoma arising in a hyperplastic polyp have been described as predominantly well differentiated histologic type, associated with intraepithelial neoplasia, gastric phenotype the same as a preexisting polyp, and a crucial role of p53 in malignant transformation [9,11,12]. Additionally, an increase in size is suggested to be a risk factor of malignancy [9]. In the case of a larger polyp measuring more than 20 mm, the incidence of adenocarcinoma transformation has been significantly increased up to 5.0% to 8.2% [9,13]. The carcinoma components are thought to arise in the surface of the polyp and extend or invade into the stalk side in a downward growth pattern [13]. According to the immunohistochemical analyses in 22 lesions of epithelial neoplasia arising in gastric hyperplastic polyps by Yao *et al*., 73.0% of neoplastic components were still classified as gastric phenotype by using the same evaluation methods as the present study [8]. As to p53, considered to be one of the most important gene products in various malignancies as well as gastric cancer, its overexpression was observed in neoplastic components associated with carcinogenesis in an early phase, whereas entirely negative in hyperplastic ones [8]. Additionally, higher Ki-67 labeling index, useful for the evaluation of proliferative activity, were also recognized only in carcinoma components, similar to the present case [8]. In this context, we believe that the current case best fits the category of carcinoma in polyp.

Surprisingly, the serum CA19-9 level before mucosal resection of the current polyp was much higher (more than 2,000 U/ml) than that after it. In addition, CA19-9 immunohistochemical expression was detected focally in this specimen. These features are consistent with CA19-9-producing gastric neoplasms, characteristics of which also are well differentiated and gastric foveolar phenotypes, but often poor prognosis with invasion depth beyond the submucosal layer [6]. There are no previous reports of CA19-9-producing *in situ *gastric cancer or CA19-9-producing gastric cancer arising in polyp such as the present case. To date only 5 early gastric cancer cases out of 28 CA19-9-producing neoplasms have been reported, and interestingly, those serum CA19-9 levels before surgery were relatively low, less than 2,000 U/ml, unlike the current one [6]. By contrast, little is clearly understood with regard to the mechanism by which CA19-9 antigens in mucins derived from gastrointestinal, colon, or pancreatic neoplasms transfer to vascular lumens. It has been reported that patients with acute cholangitis accompanied by cholestasis or with gastric ulcer after long-term administration of Sucralfate rarely have high serum CA19-9 levels [14,15]. These phenomena thus suggest that any obstructive disorder between the digestive tract lumen and mucin-secreting epithelium could induce the CA19-9 antigens to compress into the blood. As shown in Figure 2 of the present case, CA19-9 was particularly expressed on the accumulated mucin in the cystically dilated glands, reminiscent of blockade of mucin secretion. Further investigation with more clinical samples is necessary to determine how and whether or not the high serum CA19-9 levels are correlated with pathological findings and immunohistochemical expression patterns of CA19-9. While, the tumor cells can secrete CA19-9 even when the turnover of it is too rapid to accumulate and express. It is thus possible that the expression pattern of CA19-9 immunoreactivity was little significance. Nevertheless, we assume that, the serum CA19-9 level of more than 2,000 U/ml in the present case could be indicative of malignant transformation at the very least, because overexpression of CA19-9 results from a clonal growth of abnormal cells having genetic and/or epigenetic disorders [1].

On the other hand, the rate of high serum CA19-9 levels before surgery among all patients with gastric cancer is not remarkable, only 25% to 30%, and thus, the serum CA19-9 levels are not clinically important for a tumor marker, but only for a prognosis indicator [3,6,14]. In that sense, since most clinicians have never examined the serum CA19-9 levels for merely gastric polyp lesions, CA19-9-producing gastric carcinoma in polyp may be more common than generally considered.

## Conclusion

We herein reported the first case of a CA19-9-producing early well differentiated adenocarcinoma arising in gastric hyperplastic foveolar polyp. We should be aware that gastric polyp lesions might rarely produce markedly high CA19-9 antigens, possibly associated with malignant transformation.

## Consent

Written informed consent was obtained from the patient for publication of this case report and any accompanying images. A copy of the written consent is available for review by the Editor-in-Chief of this journal.

## Competing interests

The authors declare that they have no competing interests.

## Authors' contributions

XG and SY participated in conception of the idea and writing of the manuscript. XG, SY, HO, KYW, TT, AN, KK and YS performed the histopathological interpretation of the tumor tissue.

All authors have read and approved the final manuscript.
